# Varicella-Zoster Virus Infectious Cycle: ER Stress, Autophagic Flux, and Amphisome-Mediated Trafficking

**DOI:** 10.3390/pathogens5040067

**Published:** 2016-12-10

**Authors:** Charles Grose, Erin M. Buckingham, John E. Carpenter, Jeremy P. Kunkel

**Affiliations:** 1 Virology Laboratory, Children’s Hospital, University of Iowa, Iowa City, IA 52242, USA; erin-buckingham@uiowa.edu (E.M.B.); john-carpenter@uiowa.edu (J.E.C.); 2 JC Wilt Infectious Diseases Research Centre, National Microbiology Laboratory, Public Health Agency of Canada, Winnipeg, MB R3E 3L5, Canada; jeremy.kunkel@phac-aspc.gc.ca

**Keywords:** autophagy, varicella-zoster virus, cytomegalovirus, herpes simplex virus, glycoproteins, unfolded protein response, ER-associated degradation, XBP1, xenophagy

## Abstract

Varicella-zoster virus (VZV) induces abundant autophagy. Of the nine human herpesviruses, the VZV genome is the smallest (~124 kbp), lacking any known inhibitors of autophagy, such as the herpes simplex virus *ICP34.5* neurovirulence gene. Therefore, this review assesses the evidence for VZV-induced cellular stress, endoplasmic-reticulum-associated degradation (ERAD), and autophagic flux during the VZV infectious cycle. Even though VZV is difficult to propagate in cell culture, the biosynthesis of the both *N*- and *O*-linked viral glycoproteins was found to be abundant. In turn, this biosynthesis provided evidence of endoplasmic reticulum (ER) stress, including a greatly enlarged ER and a greatly diminished production of cellular glycoproteins. Other signs of ER stress following VZV infection included detection of the alternatively spliced higher-molecular-weight form of XBP1 as well as CHOP. VZV infection in cultured cells leads to abundant autophagosome production, as was visualized by the detection of the microtubule-associated protein 1 light chain 3-II (LC3-II). The degree of autophagy induced by VZV infection is comparable to that induced in uninfected cells by serum starvation. The inhibition of autophagic flux by chemicals such as 3-methyladenine or ATG5 siRNA, followed by diminished virus spread and titers, has been observed. Since the latter observation pointed to the virus assembly/trafficking compartments, we purified VZ virions by ultracentrifugation and examined the virion fraction for components of the autophagy pathway. We detected LC3-II protein (an autophagy marker) as well as Rab11 protein, a component of the endosomal pathway. We also observed that the virion-containing vesicles were single-walled; thus, they are not autophagosomes. These results suggested that some VZ virions after secondary envelopment were transported to the outer cell membrane in a vesicle derived from both the autophagy and endosomal pathways, such as an amphisome. Thus, these results demonstrate that herpesvirus trafficking pathways can converge with the autophagy pathway.

## 1. Introduction

Varicella zoster virus (VZV) is a human herpesvirus [1]. There are nine human herpesviruses, subdivided into alpha, beta, and gamma groupings. VZV is an alpha herpesvirus, as are herpes simplex virus (HSV) types 1 and 2. A representative beta herpesvirus is cytomegalovirus (CMV), and a representative gamma herpesvirus is Epstein-Barr virus (EBV). VZV has the smallest genome among all nine human herpesviruses (~124 kbp) [2]. Thus, VZV is sometimes considered to be the minimalist herpesvirus. In other words, over ~70 million years of evolution, the VZV genome lost all genes not essential for its survival [3]. As part of his review of VZV infection [1], Nobel laureate Weller noted that VZV causes the childhood disease varicella (chickenpox); in late adulthood, he proposed that the same virus that enters latency in the dorsal root ganglia after varicella reactivates to cause the disease herpes zoster (shingles). Concepts of VZV latency and reactivation have been further developed in a recent review [4]

**Figure 1:**
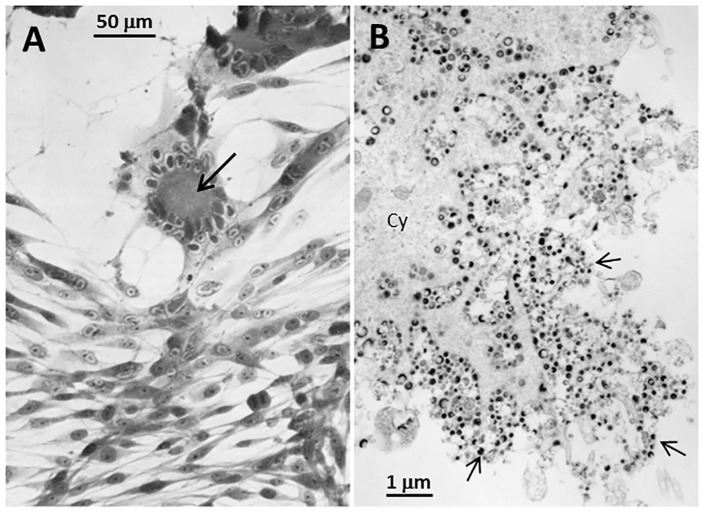
Imaging of Varicella-zoster Virus (VZV)-infected cells. (**A**) Light microscopy: Monolayer of VZV-infected cells at 72 h after infection. There are 2 foci of infection with syncytial formation (arrow). Syncytia are caused by fusion of infected cells due to the activity of fusogenic VZV glycoproteins. (**B**) Electron microscopy: Monolayer of VZV-infected cells at 96 h after infection. The viral particles (arrows) are enclosed in cytoplasmic vesicles that traffic to and fuse with the outer cell membrane. However, viral particles are not released from the outer cell membrane.

Autophagy is a well-recognized survival strategy by a stressed cell, during which misfolded or damaged proteins are engulfed within double-walled cytoplasmic organelles called autophagosomes. Autophagy is closely associated with VZV infection [5]. A vesicular skin rash is characteristic of the disease varicella; indeed, the name varicella is an irregular diminutive of the Latin word variola for an even more feared exanthem small pox. Within the cells at the base of varicella vesicles, we observed abundant numbers of autophagosomes after visualization by confocal microscopy [6]. We subsequently observed that VZV infection in cultured cells leads to both increased endoplasmic reticulum (ER) stress as well as autophagic flux, while inhibition of autophagy leads to diminished viral spread [7,8]. Unlike the closely related HSV, the VZV genome harbors no known inhibitors of autophagy, such as the HSV *ICP34.5* gene and *US11* [2]. Unlike HSV, VZV is a highly cell-associated virus that grows only in a small number of human cell lines [9]. There is no released virus, and titers of intracellular virus are invariably low [10]. Usually, 3–4 days are required before there are obvious VZV-induced cellular alterations. Since one replication cycle is estimated to be around 14 h, VZV progresses through 4–6 cycles before cytopathology is evident (Figure 1). In this review, we will re-assess the roles of ER stress and autophagy during the VZV infectious cycle. A secondary hypothesis of this review is that VZV, unlike HSV, has accommodated to autophagy without the need for any virally encoded inhibitors.

## 2. VZV Glycoprotein Biosynthesis and Expression

**Figure 2:**
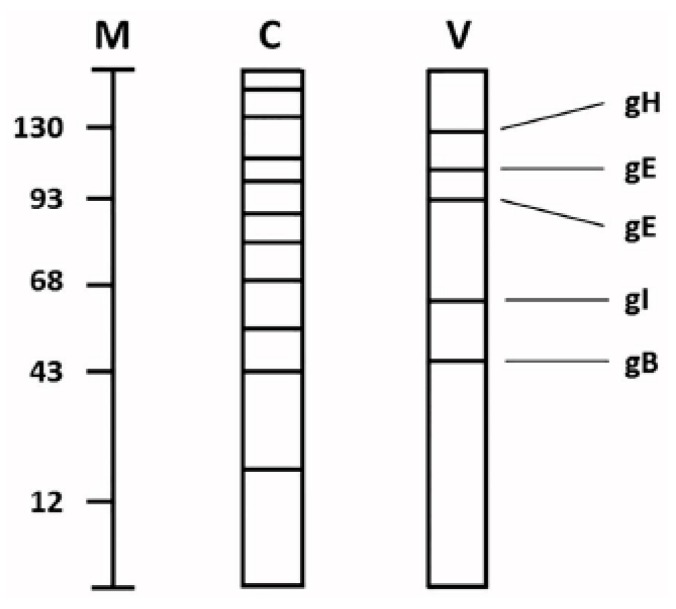
Schematic drawing of cellular and viral glycoproteins. Uninfected and VZV-infected monolayers were grown in the presence of radiolabeled glucosamine or fucose for 48 h. Thereafter, the monolayers were harvested and subjected to polyacrylamide gel electrophoresis followed by radioautography. The migration patterns of glycoproteins found in uninfected and VZV-infected monolayers are represented in lanes C and V, respectively. The current nomenclature for the VZV glycoproteins are included in the right margin. Molecular weight markers are included in lane M (kDa).

Early in VZV research, the biosynthesis of VZV glycoproteins was revealed to be highly abundant [11,12]. This observation occurred during radiolabeling experiments of VZV-infected and uninfected cells. When the cells were radiolabeled with a sugar such as glucosamine or fucose, and subjected to polyacrylamide gel electrophoresis, the profile of the glycoproteins found in the infected cells was different than the profile found in uninfected cells. We had presumed that many glycoproteins present in uninfected cells would continue to be synthesized after virus infection, but this comingling of radiolabeled cellular and viral glycoproteins generally was not found (Figure 2). The explanation for this difference was the fact that VZV glycoprotein synthesis was so abundant as to virtually exclude any further biosynthesis of cellular glycoproteins within 24 h post-infection (hpi).

**Figure 3:**
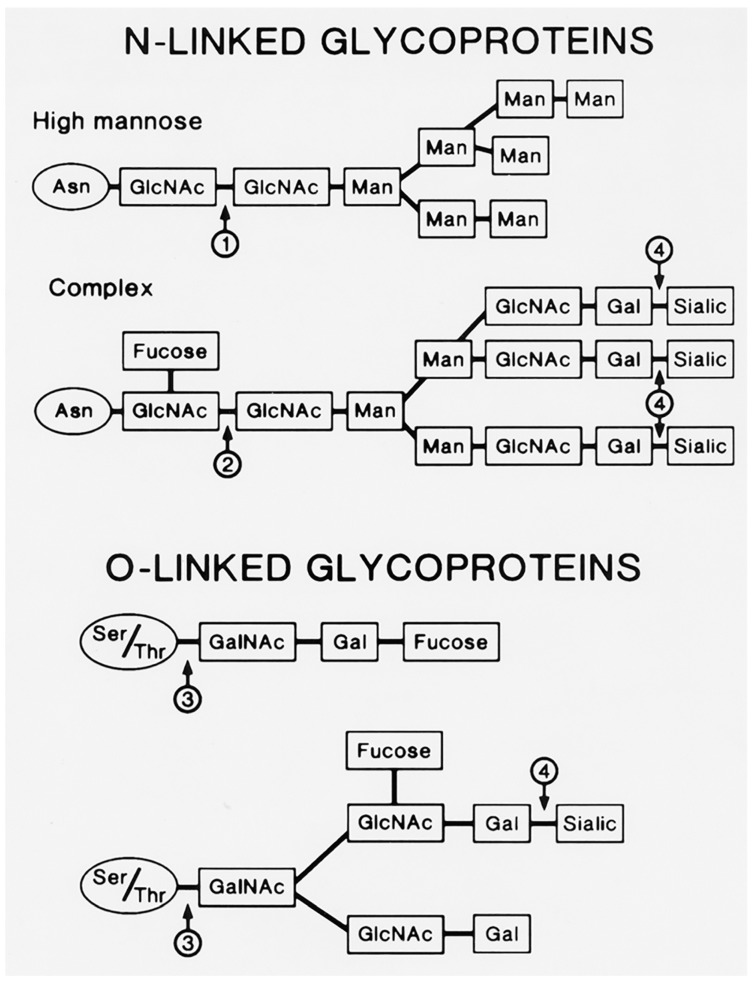
*N*-linked and *O*-linked viral glycoproteins. The viral *N*-linked glycoproteins include both high mannose and complex types. The high mannose glycoprotein shown above has already lost its three glucose residues and two of its mannose residues. The arrows indicate sites of cleavage by glycosidases: 1: Endoglycosidase H; 2: Endoglycosidase F; 3: Endo-alpha-*N*-acetylgalactosamidase; 4: Neuraminidase. GlcNAc: *N*-acetylglucosamine; Man: mannose; Gal: galactose; Sialic: sialic acid; GalNAc: *N*-acetylgalactosamine

The VZV genome encodes nine glycoproteins [13]. Several VZV glycoproteins have been characterized biochemically. The most thoroughly characterized is gE (formerly called gp98 or gpI), but others include gH, gB, and gI [14,15,16]. The predominant gE carries both *N*-linked and *O*-linked glycans (Figure 3). Glycoproteins containing *N*-glycans have the oligosaccharide *N*-glycosidically linked from the reducing end *N*-acetylglucosamine (GlcNAc) to the amide nitrogen of an asparagine residue in the polypeptide backbone. This asparagine residue is present as a part of a general consensus attachment sequence, asparagine-X-threonine/serine, where X is any amino acid except proline. *N*-glycosylation of asparagine in this sequence is also disfavored when X is a large aromatic amino acid. The parent *N*-glycan is preassembled on the luminal side of the ER membrane from 14 sugar units: 2 *N*-acetylglucosamines, 9 mannoses, and 3 glucoses (Glc_3_Man_9_GlcNAc_2_). This is then co-translationally transferred *en bloc* by a large enzyme complex to the target asparagine in the nascent polypeptide in the rough ER. The *N*-linked glycans are variously processed in the smooth ER and throughout the Golgi from the trimming (via glycosidases) of all 3 glucoses and a variable number of mannoses followed by the addition (via glycosyltransferases) of sugars such as galactose (Gal), fucose (Fuc), and sialic acid (NeuNAc) in an orchestrated manner.

Glycoproteins containing *O*-glycans have the oligosaccharide *O*-glycosidically linked from the *N*-acetylgalactosamine (GalNAc) to the hydroxyl oxygen of a threonine or serine in the polypeptide backbone (Figure 3). There is no specific consensus sequence for the attachment of *O*-linked glycans although attachment tends to occur in stretches of amino acids highly enriched in threonine and serine residues, with nearby proline residues. These clusters occur more often in less structured regions towards the *N*-termini of the polypeptides.

## 3. ER Enlargement during VZV Infection

**Figure 4:**
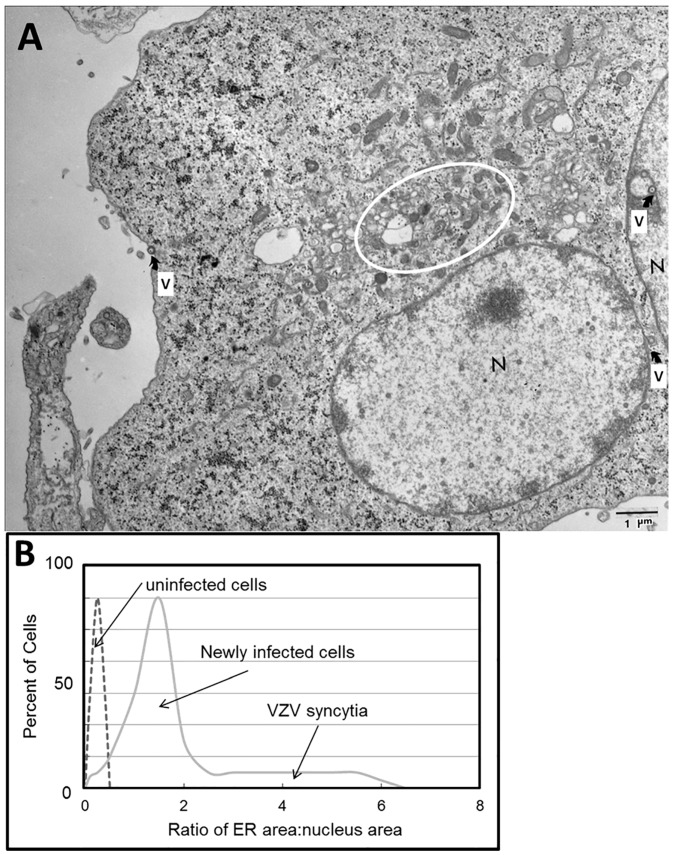
Enlarged endoplasmic reticulum (ER) area in VZV-infected cells. (**A**) Electron micrograph of infected cells. The electron micrograph shows two adjacent VZV-infected cells that have fused. Each of two nuclei is marked by the letter N. Scattered viral particles are marked by the letter V with an arrow; these include capsids within nuclei and incompletely enveloped particles in the cytoplasm. The area surrounding the ER is encircled. (**B**) Ratio of ER area to nucleus area. Note the larger ratio as the degree of infection rises from newly infected cells to advanced infection with syncytia formation.

Although unrecognized when we first began our research into the glycobiology of VZV infection, it has become apparent that VZV glycoprotein biosynthesis was associated with signs of ER stress [17]. A prominent feature of the cytopathology as seen by fluorescence microscopy is an enlarged ER [7]. When we used transmission electron microscopy (TEM) to examine VZV-infected cells, we noted that the ER possessed grossly extended folds within the infected cells. As observed by others, the expansion of the ER also includes increased spacing between the folds [18]. The enlarged ER in TEM images of VZV-infected cells also closely matched the images of dilated ER found in butyrate-treated cells, in which protein synthesis is strongly enhanced, resulting in the deposition of misfolded proteins (Figure 4A).

An enlarged ER is a characteristic of the unfolded protein response (UPR) to ER stress. In our VZV studies, we stained both VZV-infected and uninfected monolayers with DiOC6, a polar dye that localizes preferentially to ER membranes, and measured the relative size of the ER. We calculated an area for only the nucleus and the ER/nucleus complex for each image. The ER/nuclear area ratios were plotted by the number of occurrences of a given ratio. The lowest ratios were found in newly infected single cells, while the highest ratios were found in multinucleated syncytia during advanced VZV-induced cytopathology (Figure 4B). These observations indicated that the ER can be up to 10 times larger in area in VZV-infected cells compared to that in uninfected cells.

## 4. ER Stress, the Unfolded Protein Response and ER-Associated Protein Degradation

### 4.1. ER Stress and the Unfolded Protein Response

When the ER is over capacity and unable to process the load of proteins in transit, unfolded and misfolded proteins accumulate. This buildup causes ER stress and triggers the UPR. This response includes massive expansion of the ER to increase its capacity, inhibition of translation to prevent further accumulation of misfolded proteins, increased expression of chaperones, and other proteins involved in polypeptide folding, and elimination of misfolded proteins by the ERAD (ER-associated protein degradation) system.

### 4.2. ERAD and Ubiquitination

ERAD targets misfolded proteins in the ER for ubiquitination, export, and subsequent degradation by the proteasome. Misfolded proteins are detected by internal substructures such as exposed hydrophobic regions, unpaired cysteine residues, incorrect cysteine pairs (improper disulfide bonds), and immature glycans. In the latter, the lectin chaperones calnexin/calreticulin (CNX/CRT) retard the transport of immature glycoproteins and provide the opportunity for these nascent glycoproteins to reach their native conformation through the retention of misfolded glycoproteins and the de-/re-glucosylation cycle. Terminally misfolded proteins are eventually removed from this cycle by ER mannosidase I, which removes one specific terminal mannose residue from the attached *N*-glycan chain(s), and consequent recognition by EDEM1 (ER degradation-enhancing α-mannosidase-like protein 1), which helps guide the misfolded glycoproteins for export from the ER.

The targeting of abnormal or short-lived proteins for degradation through the modification of proteins with ubiquitin is an important cellular mechanism. Ubiquitination in the ERAD pathway requires at least three classes of enzymes: (E1) ubiquitin-activating enzymes, (E2) ubiquitin-conjugating enzymes, and (E3) ubiquitin-protein ligases. The E1 ubiquitin-activating enzymes hydrolyze adenosine triphosphate (ATP) and form a high-energy thioester linkage between the *C*-terminus of ubiquitin and a cysteine residue in the active site of the enzyme. The ubiquitin is thus activated and passed to a cysteine in E2 ubiquitin-conjugating enzymes. The E3 ubiquitin-protein ligases bind to misfolded proteins and align the proteins with E2 enzymes that have been loaded with ubiquitin via a structurally conserved binding region. This imparts substrate specificity to the E2s, and ubiquitin is transferred from the E2s to lysine residues of the misfolded proteins in isopeptide bonds.

A given cell type usually contains only a few forms of E1s, a larger diversity of E2s, and an abundant variety of E3s. Each type of E2 can associate with many different E3s, the latter of which are responsible for substrate recognition and binding. E3 enzymes are thus the mechanisms of substrate specificity in proteasomal degradation. Following successive additions of ubiquitin molecules to Lys48 residues of the previously attached ubiquitin, a polyubiquitin chain is formed. The resulting polyubiquitinated protein is specifically recognized by the proteasome and targeted for destruction. The polypeptide chain is fed into the central chamber core region of the proteasome which contains the proteolytically active sites. Here, ubiquitin is cleaved by deubiquitinating enzymes before terminal digestion. Since ubiquitination takes place during the translocation event, these two steps are very closely associated. However, the proteasomal degradation actually takes place in the cytoplasm.

### 4.3. XBP1 and CHOP

In addition, we have previously shown that the X-box binding protein 1, or XBP1, is detected during VZV infection, a sign of the UPR as a consequence of ER stress [19,20]. XBP1 mRNA is upregulated by the ER stress regulator protein ATF6. Another ER stress sensor protein, inositol-requiring element 1 (IRE1), which releases glucose-regulated protein 78/immunoglobulin heavy-chain-binding protein (GRP78) to support protein folding under high protein transit conditions, then becomes available for an alternative splicing of XBP1. This frameshift leads to a basic leucine zipper (bZIP) transcription factor motif in the C terminus of the translated XBP1 protein. The unspliced XBP1 is 261 residues in length, while the spliced variant is 115 amino acids longer. The alternatively spliced higher-molecular-weight form, XBP1(s), was readily visible at both 72 and 96 hpi, and first became detectable at 48 h hpi [7]. These results demonstrated unequivocally another distinctive sign of a VZV-induced ER stress response—that the IRE1 arm of the UPR was upregulated in VZV-infected cells.

C/EBP-homologous protein, CHOP or GADD153 (growth arrest and DNA damage 153), is another protein induced under conditions of severe ER stress by XBP1 and ATF4 (another UPR component). The 29 kDa CHOP is a member of the C/EBP family of transcription factors. CHOP was upregulated in VZV-infected monolayers incubated for 48 h, in concert with the appearance of the spliced form of XBP1 [7]. The parallel results for XBP1 and CHOP suggested that ER stress conditions became extreme as most of the cells became infected. Evidence for an enlarged ER as well as the formation of these proteins during VZV infection led us to the hypothesis that ER stress would facilitate increased ERAD.

## 5. VZV-Induced ERAD Microarray Analysis

**Figure 5:**
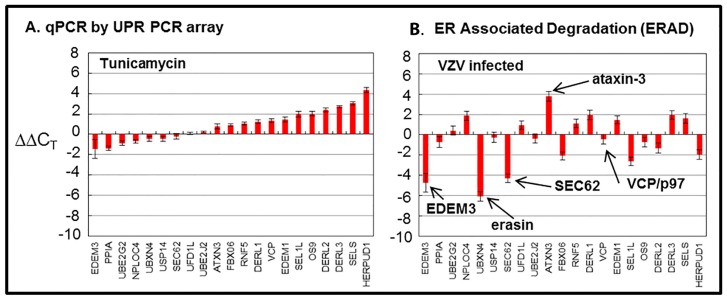
Endoplasmic-reticulum-associated degradation (ERAD) microarray analysis. Human fibroblast cells were grown in tissue culture plates, then treated with tunicamycin (TM), an *N*-glycosylation inhibitor, or infected with VZV. Subsequently, RNA was processed and gene transcription profiles were graphed for tunicamycin-treated (**A**) and VZV-infected cell samples (**B**). Arrows in B point to proteins of interest in these analyses.

Because previous findings suggested ER stress during VZV infection, we carried out a microarray analysis to assess ERAD [21]. Treatment of uninfected cells with tunicamycin was used as a control for this experiment, which was expected to elicit a similar or concordant ERAD response. The following 21 genes were surveyed in the array from the Qiagen Company (Figure 5).

### 5.1. EDEM3, also known as ER Degradation-Enhancing Alpha-Mannosidase-Like Protein 3

This protein has 931 amino acids [22]. The *N*-terminal residues (amino acids 60–499) resemble a class 1 alpha-1,2-mannosidase, while a *C*-terminal region contains a protease-associated motif (amino acids 686–780). There is a signal sequence at the *N*-terminus and an ER-retrieval signal (KDEL) at the *C*-terminus. EDEM3 is found in the ER lumen. As an example of its activity, EDEM3 interacts with the alpha 1-antitrypsin protein variant called null Hong Kong (NHK). EDEM3 increases the ERAD of the NHK glycoprotein. EDEM3 does not affect the degradation of the NHK protein when all three *N*-linked glycosylation sites are removed. These results indicate that increased ERAD of the NHK protein is related to the mannose trimming from the *N*-glycans. These results confirm the alpha-1,2-mannosidase activity of EDEM3.

### 5.2. PPIA, also known as Peptidyl-Prolyl Isomerase A and Cyclophilin A (CypA)

This protein regulates many biological processes, including intracellular signaling, transcription, inflammation, and apoptosis by catalyzing the cis-trans isomerization of proline imidic peptide bonds. CypA is one member of a family of cyclophilin proteins, which was first identified as a target of the immunosuppressive cyclosporine, used in the treatment of human diseases. As an example of its activity, CypA plays a variable role during viral infection [23]. CypA is important for successful replication of human immunodeficiency virus ( HIV) and hepatitis viruses B and C. CypA is packaged into HIV particles and therefore is essential for HIV assembly. In contrast, CypA over-expression restricts the replication of influenza A M1 protein by accelerating its transport via ERAD.

### 5.3. UBE2G2, also known as Ubiquitin Conjugating E2 Enzyme G2 and Human Homolog of Yeast Protein Ubc7

This protein is an ER membrane-bound E2 ubiquitin-conjugating enzyme, which pre-assembles Lys48-linked polyubiquitin chains and then transfers the preassembled chain to a substrate dependent on interaction with an E3 ligase enzyme [24]. This protein has two alternatively spliced transcript variants encoding distinct isoforms and is widely expressed with high expression seen in adult muscle. UBE2G2 is known to interact with the E3 ubiquitin-ligases gp78, SEL1L (see below), and DERL3 (see below), all of which contain a RING (Really Interesting New Gene) motif (see below). UBE2G2 and UBE2J2 (see below) are key E2 enzymes involved in ERAD. As an example of its activity, the knockout of the UBE2G2 gene conferred strong protection against cell death induced by the West Nile virus. However, knockout did not block virus replication.

### 5.4. NPLOC4, also known as Nuclear Protein Localization Protein 4 and Human Homolog of Yeast Protein NPL4

This protein is a ubiquitin recognition factor protein which resides on the cytosolic side of ER membrane and forms a complex with the ubiquitin fusion degradation protein 1 (UFD1L, see below) and the valosin-containing protein (VCP, see below), also called p97 [25]. A deficiency in any of the three members of the VCP/p97-UFD1L-NPLOC4/NPL4 complex leads to a decrease in degradation of a target protein in the ubiquitin-proteasome pathway. NPLOC4 contains a zinc finger domain (see RNG5 below).

### 5.5. UBXN4, also known as Ubiquitin Regulatory Domain N4, UBX Domain-Containing Protein 4, and UBX Domain-Containing 2 (UBX D2), and Erasin

This protein is an integral ER membrane protein [26]. Erasin binds valosin-containing protein (VCP) and thereby promotes ERAD. Erasin is composed of 508 amino acids, which includes an *N*-terminal coiled-coil domain, a central UBX domain, and a *C*-terminal hydrophobic domain. Erasin is oriented in the ER membrane with both its *N*- and *C*-termini facing the cytoplasm. Erasin binds VCP via its UBX domain, which is structurally very similar to ubiquitin and found in ubiquitin-regulatory proteins, which are members of the ubiquitination pathway. When erasin levels are reduced by transfection with specific siRNA, levels of ERAD are reduced.

### 5.6. USP14, also known as Ubiquitin Specific Peptidase 14 and tRNA-Guanine Transglycosylase (TGT)

This protein has been characterized with alternate transcriptional splice variants, encoding different isoforms, and is one of three proteasome-associated deubiquitinating enzymes [27]. USP14 is located in the cytoplasm and acts upon ubiquitin-cyclin B conjugates that carry more than one ubiquitin modification or chain. USP14 removes successive chains from cyclin B, until only a single chain is left. The final effect is a reduction of degradation.

### 5.7. SEC62, also known as Human Homolog of Yeast Protein and Human Homolog of Drosophila Translocation Protein 1 (Dtrp1)

This protein is a 30 kDa integral membrane protein with two transmembrane domains [28]. Both the *N*-terminus and the *C*-terminus face the cytoplasm. The *N*-terminus of SEC62 interacts with the *C*-terminus of SEC63. Together with SEC61 they form the ribosome-free SEC61-SEC62-SEC63 (or SEC61) complex. The SEC61 complex is the central component of the post-translational translocation apparatus for polypeptides across the ER membrane into the luminal compartment for further processing and folding. Depletion of SEC62 leads to a defect in ER translocation of the secretory proteins.

### 5.8. UFD1L, also known as Ubiquitin Fusion Degradation Protein 1-Like protein and Human Homolog of Yeast Protein

As described above, this protein forms a complex with two other proteins called NPLOC4/NPL4 and VCP/p97 on the cytosolic side of ER membrane [29]. This complex segregates and transports polyubiquitinated misfolded proteins for subsequent degradation by the proteasome in the ERAD pathway. Prolonged ER stress represses UFD1L expression, an effect that leads to a delay in the cell cycle. In turn, this delay in progression through G1 facilitates the clearance of misfolded proteins. A deficiency in any of the three members of the VCP/p97-UFD1L-NPLOC4/NPL4 complex leads to decreased degradation of target proteins.

### 5.9. UBE2J2, also known as Ubiquitin-Conjugating E2 Enzyme J2 and Human Homolog of Yeast Protein Ubc6

This tail-anchored membrane protein is an E2 enzyme that mediates ubiquitination of proteins mainly through lysine 48 of ubiquitin [30]. Like UBE2G2 described above, UBE2J2 is an important ERAD E2 enzyme.

### 5.10. ATXN3, also known as Ataxin 3, MJD1 (Machado–Joseph Disease), and SPA3 (Spinocerebellar Ataxia Type 3)

This protein is a 42 kDa protein with multiple domains. These include an *N*-terminal Josephin domain, a central domain containing two ubiquitin interacting motifs (UIM1 and UIM2) and a *C*-terminal domain that contains both a polyGln repeat sequence and a third UIM3 motif. The deubiquitination enzymatic activity of ATXN3 resides within the *N*-terminal Josephin domain. This domain facilitates binding and cleaving polyubiquitin chains from proteins targeted for proteasomal degradation [31]. ATXN3/SPA3 is known to interact with VCP.

### 5.11. FBXO6, also known as F-Box Only Protein 6, FBG2, FBS2, FBX6, and Human Homolog of Yeast Protein Fbx6b

This protein is a member of a family that is characterized by an approximately 40–50 amino-acid motif, known as the F-box, that mediates protein–protein interactions. F-box proteins are one of four subunits of the E3 ubiquitin ligase complex which ubiquitinates glycoproteins in the ER lumen in a phosphorylation-dependent manner, thereby directing glycoproteins toward degradation by the proteasome. FBOX6 recognizes Man and GlcNAc sugars on *N*-linked glycoproteins in the ER. This finding establishes that sugars of misfolded glycoproteins in the ER function may serve as tags for recognition for the ERAD pathway [32].

### 5.12. RNF5, also known as RING Finger 5

This protein is an ER membrane-bound E3 ubiquitin ligase. RING finger proteins have domains that are enriched for cysteine and histidine residues. These zinc-finger-type domains contain a C3HC4 amino-acid motif which binds two zinc cations. Many RING finger domains simultaneously bind E2 ubiquitin-conjugating enzymes and their substrates and hence function as E3 ubiquitin-protein ligases, targeting the substrate for degradation. As an example of its activity, RNF5 associates with and ubiquitinates a membrane associated ATG4B, which is an important LC3 protease. This interaction leads to ATG4B degradation. Thus, RNF5 activity modulates ATG4B levels and thereby affects LC3 turnover and formation of autophagosomes [33].

### 5.13. DERL1, also known as DER (Degradation in Endoplasmic Reticulum)-Like Protein 1 and Derlin 1

This protein is an ER membrane E3 ubiquitin ligase, and is part of a complex, which includes SELS (see below), SEL1L (see below), HRD1 (an E3 ubiquitin ligase), and HERPUD1 (see below. This complexmediates ERAD, which detects misfolded proteins in the ER and targets them for destruction. The deglycosylating enzyme called peptide *N*-glycanase (PNGase/PNG1) acts on misfolded *N*-linked glycoproteins [34]. In turn, DERL1 associates with the *N*-terminus of PNGase via its cytosolic *C*-terminus. This process occurs at the cytoplasmic face of the ER membrane and in the cytosol. The interaction of DERL1 with PNGase/PNG1 may feed both type I and type II ERAD [35].

### 5.14. VCP, also known as Valosin-Containing Protein, Human Homolog of Yeast CDC48 Protein, p97, and Transitional Endoplasmic Reticulum ATPase (TER ATPase)

This protein is a member of the AAA family of ATPases. Many members of this family are important chaperones that regulate folding or unfolding of substrate proteins. Although the original designation of the name valosin was incorrectly based on an artifact of purification, the name persists in the literature. VCP/p97 is one of the most abundant cytoplasmic proteins in eukaryotic cells, and a significant fraction of this is associated with ER, Golgi, mitochondrial, and endosomal membranes [36]. It functions mainly to segregate proteins from large cellular structures such as protein assemblies, organelle membranes, and chromatin, allowing the released proteins to be degraded by the proteasome.

VCP partners with diverse proteins in order to process ubiquitin-labeled proteins for ERAD. For example, VCP/p97 is a deubiquitinating enzyme in a complex with NPLOC4 (see above) and UFD1L (see above) which transports substrates from the ER to the cytoplasm and regulates polyubiquitination of translocated ER substrates. VCP/p97 also interacts with ATXN3/SPA3 (see above), another deubiquitinating enzyme. VCP and EDEM1 (see below) form a complex in the presence of DERL2 (see below). VCP also interacts with UBXN4/erasin which possesses an ubiquitin-like domain that may facilitate association with polyubiquitinated substrates and translocation. VCP/p97 is also implicated in autophagy, the process in which cellular proteins (including misfolded ones) are turned over by engulfment into double-membrane-surrounded vesicles named autophagosomes, but the role of VCP/p97 in this mechanism requires elucidation.

### 5.15. EDEM1, also known as ER Degradation-Enhancing Alpha-Mannosidase-Like Protein 1

This protein is a stress-regulated protein. The activity of EDEM1 is dependent on the proximal ER stress sensor IRE1alpha; when IRE1alpha function is decreased, EDEM1 function is decreased. As described above in Section 4.2, EDEM1 is a component of the quality control mechanism for *N*-linked glycoproteins [37]. Aberrantly glycosylated and/or misfolded glycoproteins are extracted by EDEM1 from the CNX/CRT cycle and forwarded toward ERAD. EDEM1 forms a complex with VCP/p97 (see above) in the presence of DERL2 (see below). It is also known to associate with SEL1L (see below) and OS9 (see below).

### 5.16. SEL1L, also known as Suppressor Enhancer Lin and Human Homolog of Yeast Hrd3 Protein (Hydroxymethyl-Glutaryl Reductase Degradation Protein 3)

This protein is a 794-amino-acid ER membrane-anchored protein. Expression of SEL1L is induced during the UPR triggered by the accumulation of misfolded proteins in the ER. It is a RING finger-containing E3 ubiquitin ligase and a major mediator of substrate ubiquitination during ERAD. SEL1L is part of a complex, which includes DERL1 (see above), SELS (see below), HRD1 (an E3 ubiquitin ligase), and HERPUD1 (see below). This complex mediates ERAD [38]. It interacts with the E2 ubiquitin-conjugating enzymes UBE2J2 (see above) and UBE2G2 (see above). It is also known to associate with OS9 (see below), EDEM1 (see above), EDEM3 (see above), and DERL3 (see below).

### 5.17. OS9, also known as Osteosarcoma Amplified 9, Endoplasmic Reticulum Lectin 2, and ERLEC2

This protein is an ER protein that functions as a lectin that is required for glycoprotein protein quality control and ERAD [39]. OS9/ERLEC2 recognizes the single-mannose-trimmed *N*-glycan “glycoprotein misfold” signal in the same manner as EDEM1 (see Section 4.2). OS9 associates with SEL1L (see above), EDEM1 (see above), and HRD1 (an E3 ubiquitin ligase).

### 5.18. DERL2, also known as Degradation in ER-Like Protein 2 and Derlin-2

This protein and DERL3 (see below) were discovered during a search for genes induced by ER stress [40]. DERL2 and DERL3 show approximately 75% identity. The identity between DERL1 and either DERL2 or DERL3, in contrast, is only about 30%. DERL2 and DERL3 are required for efficient ERAD. DERL2 and DERL3 are associated with VCP/p97 and EDEM1. In fact, EDEM1 and VCP/p97 form a complex only in the presence of DERL2.

### 5.19. DERL3, also known as Derlin-3 and Synoviolin 1 (SYVN1)

Like DERL1 (see above) and DERL2 (see above), this protein is an E3 ubiquitin ligase in the ER membrane which functions in ERAD to remove misfolded proteins accumulated during ER stress by retrotranslocation to the cytosol from the ER. DERL3/SYVN1 also uses the ubiquitin-proteasome system for additional degradation of misfolded proteins [40].

### 5.20. SELS, also known as Selenoprotein S, SEPS1, SELENOS, and VIMP (VCP-Interacting Membrane Protein)

This protein contains the 21st amino acid called selenocysteine (Sec; U), which is encoded by the UGA codon that normally signals termination of translation. There is a common stem-loop structure in the 3'-UTR of selenoprotein genes, the sec insertion sequence (SECIS), which recognizes the UGA triplet as a Sec codon rather than as a stop signal. The protein contains 189 amino acids with a single transmembrane domain (24 residues), a short ER luminal domain (27 amino acids) and a long *C*-terminal tail that includes the selenocysteine residue. SELS is a component of the ERAD system [41]. One pseudonym for SELS is VIMP (VCP-interacting membrane protein). It forms a complex with VCP/p97 (see above) and DERL1 (see above) and anchors it to the ER membrane. SELS/VIMP also functions as a thiol-disulfide oxidoreductase, and this presumably allows it to reduce particularly stable disulfides in ERAD substrates that were not reduced prior to retrotranslocation. Currently it is unclear how the ubiquitination machinery is recruited to this complex, but the associations between VCP, DERL1, and HERPUD1 (see below) may play distinct roles. This protein may also regulate cytokine production and thus play a key role in control of the inflammatory response.

### 5.21. HERPUD1, also known as Homocysteine-Responsive ER-Resident Ubiquitin-Like Domain Member 1 Protein and HERP

This protein consists of 391 amino acids, with one transmembrane domain. Different isoforms from multiple transcript variants are encoded by alternative splicing. The full-length nature of all transcript variants has not been determined. HERPUD1 has an *N*-terminal ubiquitin-like domain that interacts with the ERAD system, and expression from the gene is induced by UPR through an ER stress response element in its promoter region [42]. HERPUD1 is a component of a larger ER-resident complex that includes HRD1 (an E3 ubiquitin ligase), DERL1, SELS/VIMP, and VCP/p97. HERP binds directly to the E3 ubiquitin ligase; in turn, the E3 ligase is bound to DERL1, SELS, and VCP. This complex facilitates ubiquitination and retrotranslocation of ERAD substrates to avoid ER-stress induced apoptosis.

## 6. ERAD Types I and II

**Figure 6:**
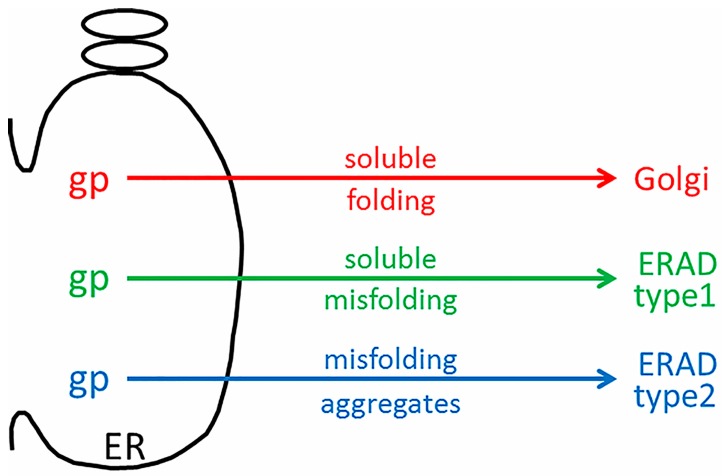
Schematic drawing of ER stress during VZV infection. During VZV infection, abundant viral glycoproteins (gp) are processed in the ER. Viral glycoproteins that are correctly folded traffic to the Golgi for further processing into complex-type glycoproteins (red arrow). During ER stress, viral glycoproteins that are soluble and incorrectly folded may exit by ERAD type I (green arrow). Viral glycoproteins that are misfolded and aggregated may exit by ERAD type II (blue arrow).

The discordant microarray results between the tunicamycin-treated “control” cultures, and the VZV-infected cultures were not expected [43]. Tunicamycin is an antibiotic that inhibits transfer of *N*-acetylglucosamine-1-phosphate from the uridine diphosphate (UDP)-*N*-acetylglucosamine donor sugar to dolichol phosphate. This prevents *N*-linked glycosylation by blocking the creation of the Glc_3_Man_9_GlcNAc_2_ oligosaccharide donor sugar which is co-translationally attached to an asparagine residue in the first step of *N*-linked glycosylation. Thus, the proper formation of *N*-linked glycoproteins is blocked, and there is a rapid accumulation of misfolded nascent glycoproteins in the ER. The tunicamycin microarray data illustrate an expanded conventional ERAD response (type I) to the accumulation of misfolded glycoproteins in the ER which is secondary to this tunicamycin block. The microarray data from VZV-infected cells did not suggest a similar high degree of conventional ERAD following misfolding of nascent VZV glycoproteins. Therefore, we formulated a new hypothesis, namely, that ER stress during VZV infection leads to an expanded ER that is able to accommodate the abundant biosynthesis and processing of VZV glycoproteins because of type II ERAD (Figure 6).

Type II ERAD is ERAD linked to autophagy, sometimes abbreviated to ER-phagy (18). When the capacity of the ER to remove misfolded proteins by type I ERAD is exceeded or when there is an accumulation of aggregated proteins, autophagy can be induced in response to this maximal ER stress. During autophagy induced by ER stress, double-membrane vesicles termed autophagosomes engulf portions of the ER with associated proteins and protein aggregates. These are delivered to the lysosome or vacuole for degradation. Autophagy thus acts as an alternative ERAD pathway, particularly for aggregation-prone substrates. One substrate for which this is well shown is the mutant aggregation-prone Z variant form of alpha1-antitrypsin (different from the soluble NHK mutant described above in the EDEM3 section). Although the Z variant is usually degraded by ERAD, it can accumulate and polymerize in the ER before being delivered. Autophagosomes likely engulf ER fragments which contain these polymerized mutant protein aggregates.

Thus, the UPR, ERAD, and ER-phagy appear to work together to expand or shrink the ER as needed, and to relieve ER stress by refolding or degrading damaged proteins. However, this requires fine control. ERAD is modulated by the load of misfolded protein in the ER. An example of such control is the HRD1 “dislocon complex”. This complex, comprised of HRD1 (E3 ubiquitin ligase), SEL1L (an ER membrane-anchored RING finger-containing E3 ubiquitin ligase), DERL1 (E3 ubiquitin ligase), and several other luminal, membrane, and cytosolic accessory proteins, conveys ERAD substrates to the ER membrane and ultimately to cytosolic proteasomes. This multiprotein complex contains HERPUD1, which possesses a half-life 50–200 times shorter than most of the HRD1 translocation machinery components and other conventional ER-resident proteins. The presence of misfolded or aggregated proteins continues to maintain fully assembled and functional HRD1 dislocons. When not required, HRD1 dislocon complexes are disassembled, and HERPUD1 is rapidly turned over by the ubiquitin-proteasome system. However, if the flux of such proteins overwhelms the otherwise rapid turnover of HERPUD1, then an alternative mechanism, ER-phagy, is required.

## 7. Autophagic Flux during VZV Infection

The 2016 Nobel Prize for Medicine or Physiology was awarded to Professor Yoshinori Ohsumi for his pioneering research on mechanisms of autophagy; his research was carried out mainly in yeast [44,45]. Autophagy in human tissues is induced through the inhibition of the mammalian target of rapamycin (mTOR) under various conditions, e.g., starvation or infection of cells [46,47]. We have previously shown in several published articles that autophagy is induced following VZV infection in humans [8,48,49]. VZV infection in humans leads to a distinctive exanthem (skin rash) where final assembly of enveloped virions takes place. The infected keratinocytes removed from these rashes display scores of autophagosomes [50]. Their number have been carefully enumerated by our laboratory, after examining skin cells from varicella and herpes zoster lesions by high resolution confocal microscopy. VZV-infected skin cells have even more autophagomes per cell than VZV-infected monolayers (100 vs. 20–30/cell).

After induction of autophagy, the mTOR substrate complex (ULK1,ULK2, ATG13, FIP200, and ATG101) translocates from the cytosol to the ER, where the complex associates with vacuole membrane protein 1 (VMP1). This interaction activates the ER localized class III phosphatidylinositol-3-kinase (PI(3)K) to generate phosphatidylinositol 3-phosphate (PI3P). This PI(3)K is a positive regulator of autophagy; the kinase complex includes VPS34 (PIK3C3 gene product), VPS15 (PIK3R4 gene product and p150), Beclin-1, and ATG14. The kinase complex recruits PI3P binding proteins including WIPIs on the ER membrane. WIPIs are WD-repeat proteins interacting with phosphoinositides, members of a larger PROPPIN family that bind to both PI(3)P and the vacuolar lipid PI(3,5)P2.

**Figure 7:**
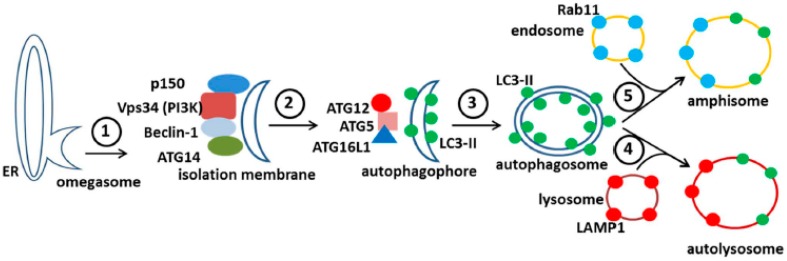
Diagram of the autophagy pathway in mammalian cells. The major features in the autophagic pathway are illustrated, including the abbreviations of important autophagy proteins (atg). Omegasomes form from ER membranes (step 1) and develop into isolation membranes (step 2). LC3 is lipidated to form LC3-II and is incorporated into autophagophores, which close to form double membraned autophagosomes (step 3). Note that the LC3-positive autophagosome can fuse with either an endosome (step 5) or a lysosome (step 4). This results in the formation of either an amphisome (with Rab11 (blue) and LC3-II (green)), or an autolysosome (with LAMP1 (red) and LC3-II (green)).

The above events initiate the nucleation and the remodeling of the phagophore (preautophagosomal) membranes (Figure 7). At the same time, the *C*-terminus of the microtubule-associated protein 1 light chain 3, abbreviated LC3, is cleaved by the protease Atg4 to produce LC3-I, an 18 kDa protein that is distributed throughout the cytoplasm. During the subsequent stage of autophagosome formation, the double-membraned vacuolar structure elongates and closes, a step requiring the ATG12-ATG5 conjugate together with its partner ATG16LI. The latter complex also effects the conjugation of LC3-I with phosphatidylethanolamine (PE) to create the LC3-II isoform, which migrates slightly faster by SDS-PAGE (16 kDa) [51]. Lipidated LC3-II is attached to both the inner and outer membranes of the maturing autophagosome (Figure 7). Thereafter, autophagosomes are easily identified by immunostaining of the LC3-II proteins with fluorescent probes; the distinctive autophagosomes are usually described as puncta. Subsequently, autophagosomes fuse with lysosomes to form autolysosomes. The cargo within the autophagosome is degraded by the lysosomally derived hydrolases. LC3-II isoforms located on the inner membrane are degraded while LC3-II isoforms on the outer membrane are recycled after delipidation by ATG4. At this point, in the absence of LC3-II, the autolysosome becomes a lysosome and is no longer detectable with LC3 antibody probes. As an alternative to fusion with a lysosome, an autophagosome can also fuse with an endosome to form an amphisome. The completion of this cycle is termed autophagic flux.

**Figure 8:**
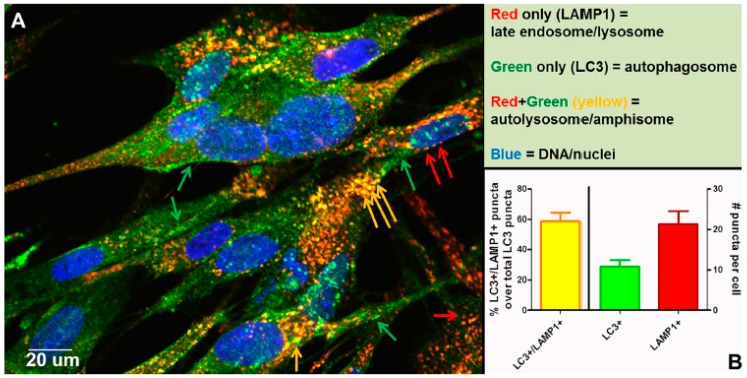
Completion of autophagic flux during VZV infection. At 72 h post infection (hpi), infected cells were fixed with 2% paraformaldehyde and then processed for examination by confocal microscopy. Monolayers were incubated in primary antibodies: rabbit anti-LC3B and mouse anti-LAMP1. Monolayers were then incubated in the following antibodies: goat-anti-mouse-AlexaFluor 546, goat-anti-rabbit-AlexaFluor 488, as well as H33342. (**A**) Micrograph of a representative 400× image. Red arrows point to late endosomes/lysosomes (LAMP1+), green arrows point to autophagosomes (LC3+), and yellow arrows point to autolysosomes/amphisomes (LC3+/LAMP1+) (see Figure 7). (**B**) Quantitation of puncta. Puncta from many images were quantitated (>30 cells). The chart shows the percent puncta that were both LC3+ and LAMP1+ over total LC3+ puncta (left Y-axis), as well as average numbers of LC3+ puncta and LAMP1+ puncta per cell (right Y-axis).

Other than autophagic flux, an alternative explanation for the increased number of autophagosomes following VZV infection is a block in flux, which prevents the transition from autophagosome to lysosome or amphisome. To disprove the presence of a block, we performed an experiment to document that autophagic flux was easily detected in VZV-infected monolayers that were labeled with antibodies against LC3 and LAMP1, as shown by colocalization and quantitation of puncta that were both LC3+ and LAMP1+ (Figure 8). As per guidelines of Klionsky et al. [52,53], we had documented previously an absence of a block in autophagic flux in VZV-infected monolayers by three other methods: (i) immunoblotting stress inducible p62/SQSTM1 protein levels; (ii) measuring degradation of radiolabeled long-lived proteins; and (iii) visualizing change in color of the mRFP-GFP tandem fluorescent tagged LC3 plasmid [48,49].

## 8. Amphisomes and Virus Trafficking Pathways

**Figure 9:**
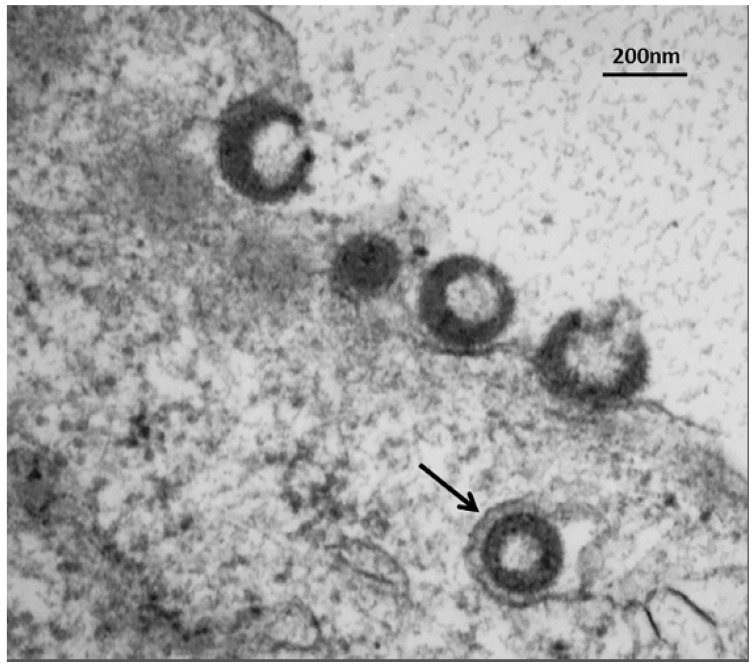
Trafficking of VZV particles to the cell surface. The electron micrograph shows one VZV particle within a single-walled cytoplasmic vesicle (arrow) at 72 hpi. Four recently egressed viral particles are also visible at the outer cell membrane, some of which may be light particles (viral envelopes without a capsid). These VZV particles cannot be input virus from the inoculum, since prior electron microscopy studies have shown that viral particles are rarely detectable at or near the outer cell membrane for the first 24 hpi, because the titer of input virus is so low [56]. Viral particles only become easily detectable by electron microscopy after formation of progeny virus.

In a recent publication, we demonstrated a convergence of autophagy and endosomal pathways in the late phase of the VZV infectious cycle [54]. We showed that some VZ virions were enclosed within single-walled vesicles with attributes of an amphisome. To obtain this data, we first defined the optimal purification protocol for VZ virions. We tested several density gradient media and discovered that a combination of potassium tartrate and glycerol led to the least amount of de-envelopment of complete virions after high-speed centrifugation. Our final protocol included two sequential sedimentations in these density-viscosity gradients. Of great interest, virologists studying the structure of enveloped RNA viruses also consider potassium tartrate gradients to be the preferred media for purification [55]. As shown in Figure 7, an amphisome is formed by fusion of an autophagosome with an endosome; an amphisome contains two distinctive markers: LC3 (autophagy pathway) and Rab11 (endosomal pathway). In other words, not all autophagosomes fuse with lysosomes. Our data suggest that during or immediately following secondary virion envelopment in the virus assembly compartment, some virions are enclosed and transported to the cell surface within a single-walled vesicle with properties of an amphisome (Figure 9). Therefore, any enhanced production of autophagosomal membranes would enable increased trafficking of VZ virions in amphisomes or an amphisome-like vesicle. We did not find any evidence for xenophagy of VZV particles after examination of over 100 electron micrographs of VZV-infected cells. Further research will be required to define more detailed contributions of the autophagy pathway to VZV assembly and trafficking.

## 9. Basal versus Induced Autophagy

As noted in the above sections, VZV infection induced abundant autophagosome formation beyond basal levels both in skin cells from humans with varicella skin vesicles and in infected cultured cells. A recent investigation of HSV-2 infection has provided additional insight into basal versus induced autophagy following virus infection [57]. The HSV-2 genome, similar to the HSV-1 genome, contains the autophagy inhibitory gene *ICP34.5*; the HSV-2 *ICP34.5* gene codes for four different splice variant derived proteins [58,59]. As anticipated, HSV-2 infection did not induce autophagy after infection. However, when HSV-2 was grown in murine cells lacking the essential autophagy gene *ATG5*, the titer fell to 1000 plaque forming units per mL as compared with a titer of >1 million plaque forming units per mL in murine cells containing the *ATG5* gene. As a control experiment, the rates of virus entry were compared and found to be comparable in the cells with and without the *ATG5* gene. As a second control experiment, different strains of HSV-2 were tested, and the results showed the same differential titers between growth in cells with and without the *ATG5* gene. These results suggested that basal levels of autophagy were essential for the HSV-2 infectious cycle, even in a herpesvirus expressing the ICP34.5 protein. The authors speculated that the autophagy pathway may be required for assembly or transport of HSV-2 viral particles [57].

## 10. Conclusions

As noted, the VZV genome is the smallest genome among the human herpesviruses. Unlike other human herpesviruses such as CMV with a much larger genome, VZV appears to have few genes that interact with ERAD [60,61]. Unlike HSV type 1 with its slightly larger genome including an anti-autophagy gene called *ICP 34.5*, VZV has no known functional *ICP34.5* homolog that inhibits autophagy [62]. Instead, with an economy of genes, VZV appears to navigate through a cell by subtly co-opting cellular processes for its own benefit. One example appears to be a convergence of autophagy and endosomal pathways in order to transport viral particles following secondary envelopment. Overall, the effects of ER stress and subsequent autophagy likely prolong the lifespan of a VZV-infected cell and thereby allow for greater production of infectious virus.
